# A novel frame-shift deletion in *FANCF* gene causing autosomal recessive Fanconi anemia: a case report

**DOI:** 10.1186/s12881-019-0855-2

**Published:** 2019-07-09

**Authors:** Soheila Zareifar, Hassan Dastsooz, Mahdi Shahriari, Mohammad Ali Faghihi, Golsa Shekarkhar, Mohammadreza Bordbar, Omid Reza Zekavat, Nader Shakibazad

**Affiliations:** 1 Hematology Research Center, Shiraz University of Medical Sciences, Shiraz, Iran; 2 Italian Institute for Genomic Medicine (IIGM), University of Turin, Turin, Italy; 3 Division of Pediatric Hematology and Oncology, Department of Pediatric, Shiraz University of Medical Sciences, Shiraz, Iran; 4 Center for Therapeutic Innovation, Department of Psychiatry and Behavioral Sciences, University of Miami Miller School of Medicine, Miami, USA; 5 Molecular Pathology Center, Shiraz University of Medical Sciences, Shiraz, Iran; 6 Pediatric Hematology and Oncology, Bushehr University of Medical Sciences, Bushehr, Iran

**Keywords:** *FANCF*, Novel mutation, NGS, Autosomal recessive Fanconi Anemia

## Abstract

**Background:**

Fanconi anemia (FA) is a heterogeneous genetic disorder characterized by congenital anomalies, early-onset bone marrow failure, and a high predisposition to cancers. Up to know, different genes involved in the DNA repair pathway, mainly FANCA genes, have been identified to be affected in patients with FA.

**Case presentation:**

Here, we report clinical, laboratory and genetic findings in a 3.5-year-old Iranian female patient, a product of a consanguineous marriage, who was suspicious of FA, observed with short stature, microcephaly, skin hyperpigmentation, anemia, thrombocytopenia and hypo cellular bone marrow. Therefore, Next Generation Sequencing was performed to identify the genetic cause of the disease in this patient. Results revealed a novel, private, homozygous frameshift mutation in the FANCF gene (NM_022725: c. 534delG, p. G178 fs) which was confirmed by Sanger sequencing in the proband.

**Conclusion:**

Such studies may help uncover the exact pathomechanisms of this disorder and establish the genotype-phenotype correlations by identification of more mutations in this gene. It is the first report of a mutation in the FANCF gene in Iranian patients with Fanconi anemia. This new mutation correlates with a hematological problem (pancytopenia), short stature, and microcephaly and skin hyperpigmentation. Until now, no evidence of malignancy was detected.

**Electronic supplementary material:**

The online version of this article (10.1186/s12881-019-0855-2) contains supplementary material, which is available to authorized users.

## Background

Fanconi anemia (FA) is a clinically and genetically heterogeneous uncommon autosomal recessive disorder with hallmarks of congenital malformations, early-onset bone marrow failure, and a high susceptibility to malignancies due to genomic instability [1, 2]. FA is resulted from disease-causing mutations in *FANC* genes. *FANC* genes encode a group of proteins, which act in the pathway of DNA-damage repair along with other proteins. Up to now, 22 *FANC* genes have been identified among which *FANCA* mutations, known as hyper-mutable genes, have been reported to be the most common genetic causes of FA patients.

The confirmation of FA diagnosis in a proband should be considered with the following examination: Firstly, cytogenetic examination with increased levels of chromosomal breaks and radial formation on lymphocytes after exposure to Diepoxybutane (DEB) or Mitomycin C (MMC) and secondly, identification of pathogenic mutations in one of the 22 FA genes [3–5].The aim of our study was to discover the mutated genes in an affected Iranian patient with FA using Next Generation Sequencing (NGS).

## Case presentation

**Fig. 1:**
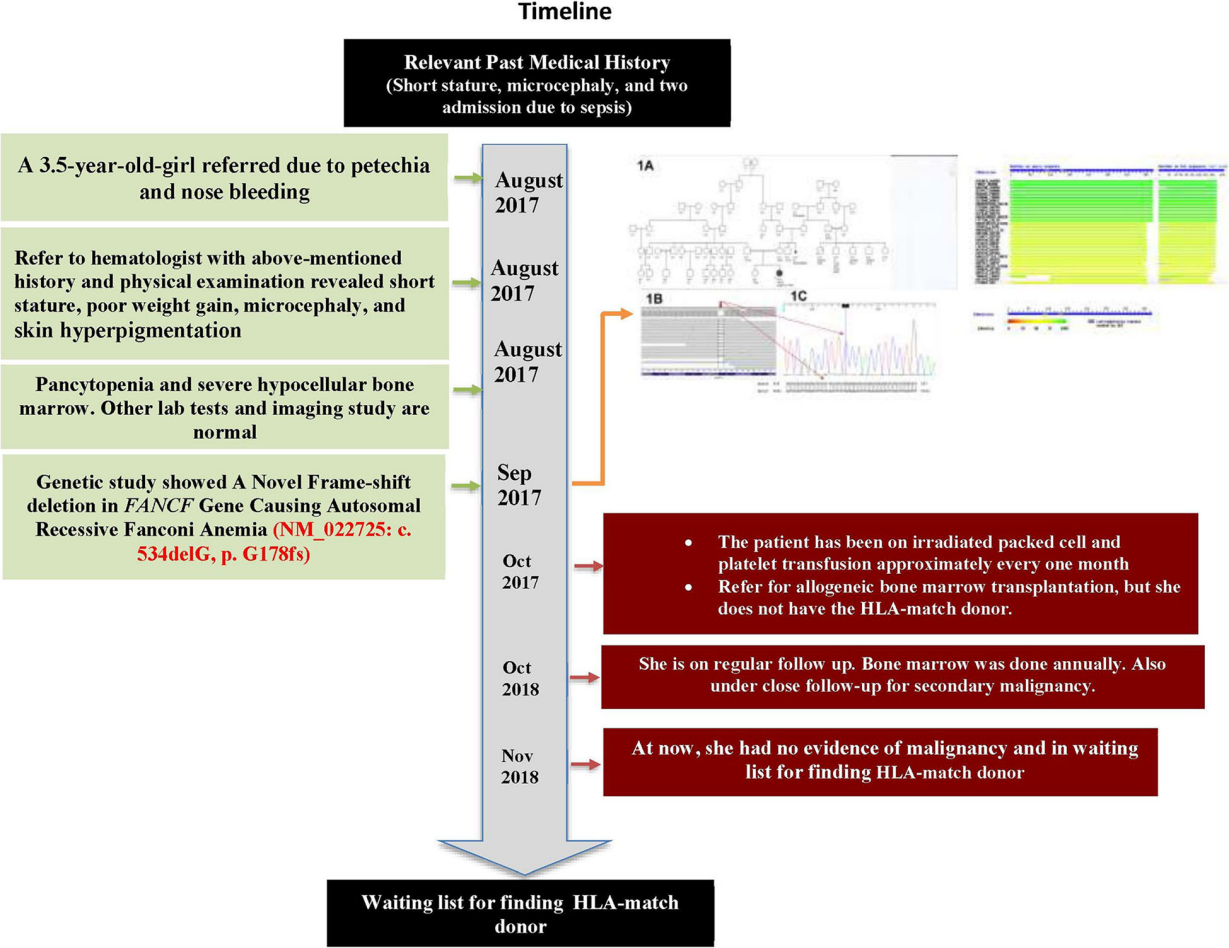
Information from this case report organized into a timeline figure

**Fig. 2:**
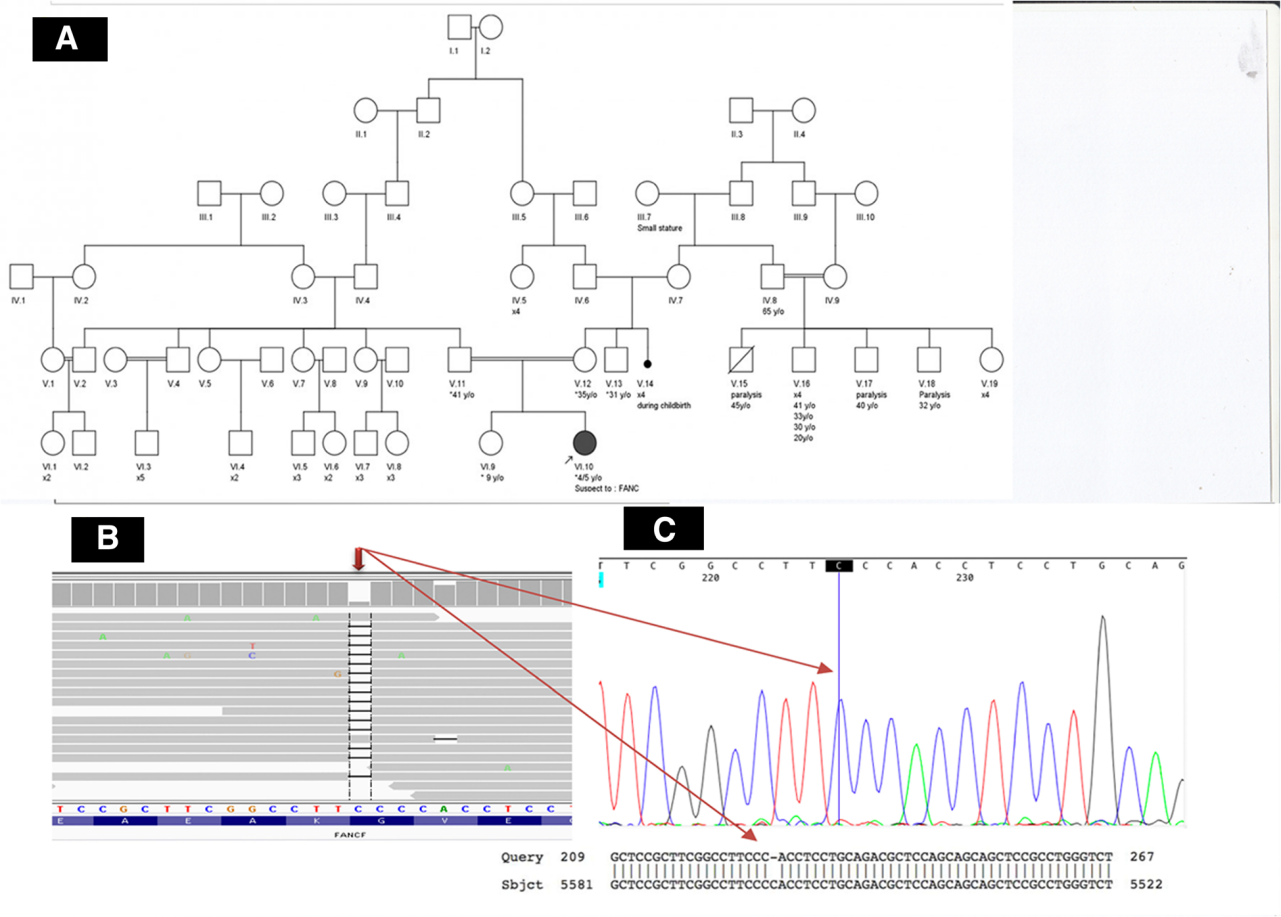
**a**: Pedigree, **b**: NGS data on IGV, and **c**: Sanger sequencing chromatogram. In the pedigree, proband is shown as a product of consanguineous marriage and the first case affected by FA in this family. Bam file data of the patient show the homozygous deletion (left). Sanger sequencing and NCBI blast confirmed this homozygous deletion (right)

A 3.5-year-old girl, Caucasian, who was a product of a consanguineous marriage (first-degree cousins, Fig. 1 (timeline of case presentation) and Fig. 2a) was registered in our department due to petechia and nose bleeding. She was born at 37 weeks and 5 days of normal vaginal delivery. Her birth weight, head circumference, and height were 2.8 kg, 32 cm, and 46 cm, respectively. Although her development was in the normal range, the growth chart revealed that her height and head circumference were below the third percentile line, and her weight was around the 5th percentile line. In addition, she took zinc supplements due to her short stature. On physical examination, short stature, poor weight gain, microcephaly (head circumference; 46 cm) and skin hyperpigmentation were detected. She had a history of two-time hospital admission due to pneumonia.

Due to petechia, complete blood count was performed and the results identified anemia (Hb: 7.2 g/dL), leukopenia (WBC: 1.5 × 10^3^/μL with an absolute neutrophil count of 455), and thrombocytopenia (Platelet count: 9 × 10^3^/μL). Other laboratory and imaging findings, including C3, C4, ANA, dsDNA, CH50, Immunoglobulin level, TORCH study, metabolic panel, biochemistry studies, abdomen and pelvic sonography, brain MRI, lumbosacral and both hands X-ray, and echocardiography were normal. In addition, bone marrow aspiration and trephine biopsy revealed presented megakaryocyte and moderate to severe hypocellular bone marrow.

The patient had been on irradiated packed cell and platelet transfusion approximately every one month. She was a candidate for allogeneic bone marrow transplantation, but she did not have the HLA-match donor. She was under regular follow-up and occasionally referred due to epistaxis or pallor, and received irradiated packed cell and platelet.

### Next generation sequencing (NGS)

Written informed consent was obtained from the parents. Whole blood samples were collected using EDTA tubes. Genomic DNA was prepared from peripheral leukocytes of the patient using the QIAamp DNA Blood Mini Kit (Qiagen, Germany) and then NanoDrop (ND1000, USA) was used to measure DNA concentration.

NGS covering immunological and hematological disorders was carried out on Illumina NextSeq500 machine to the sequence close to 100 million reads. Bioinformatics analysis of the sequencing results was performed using BWA aligner [6], GATK [7] and annovar [8] as well as different databases and bioinformatics software such as REVEL, MCAP, ESP6500,1000G, Clinvar, CADD-Phred, SIFT, PolyPhen, GERP, PhastCons, LRT, Mutation Assessor, Mutation Taster, phyloP46way_placental, phyloP100way_vertebrate, SiPhy_29way, FATHMM_pred, RadialSVM, ExAC. Kaviar, GME, gnomAD.

### Sanger sequencing

To confirm the novel identified mutation, we performed Sanger sequencing of the genomic DNA from the proband. For this test, PCR was carried out for the patient’s DNA using the following primers: F-FANCF: CGCTGGGAGATTGACATG and R-FANCF: GACCCCAGTCTGTTAGCA (PCR product: 978 bp) to amplify a mutated region of *FANCF.* Then, Sanger sequencing was used to sequence amplified DNA with both forward and reverse primers using ABI BigDye Terminator Cycle Sequencing Kit (Applied Biosystems®, USA). The analysis of Sanger sequencing data was performed with NCBI BLAST and Codon Code Aligner software. Multiple sequence alignment analysis was performed using the SIB BLAST+ Network Service From (https://web.expasy.org/blast/) to compare the amino acid sequence of human FANCF proteins with other proteins across different Kingdoms. STRING (STRING: functional protein association networks, https://string-db.org), tool and KEGG database (KEGG: Kyoto Encyclopedia of Genes and Genomes, http://www.genome.jp/kegg/) were also used to explain the FA pathway and its protein network.

### Cytogenetic examination

Owing to suspicion to inherited bone marrow failure, chromosomal study with MMC on the peripheral blood lymphocyte culture of the proband was requested. To evaluate the types and rates of breakages and rearrangements in the chromosomes of the cells in the proband, GTG banding and the chromosome breakage test were performed on the patient’s blood sample. The blood sample was then cultured and treated with different concentrations of MMC**.**

NGS revealed a novel, private, homozygous, frame-shift deletion mutation in the *FANCF* gene (FANCF-201, ENST00000327470.4, NM_022725: exon1, c. 534delG, p. G178 fs, position 22,625,277 on chromosome 11). Using Sanger sequencing, the mutation was confirmed in the proband as homozygous (Fig. 2b and c). The identified mutation has not been reported yet in any database of genomic variants including ESP6500, 1000 Genome Project, ExAC, Kaviar, GME, gnomAD, and our internal database (Bayan Gene), confirming the novelty of mutation. This is the first report of *FANCF* mutation in Iranian patient affected with autosomal recessive FA, complementation group F.

**Fig. 3:**
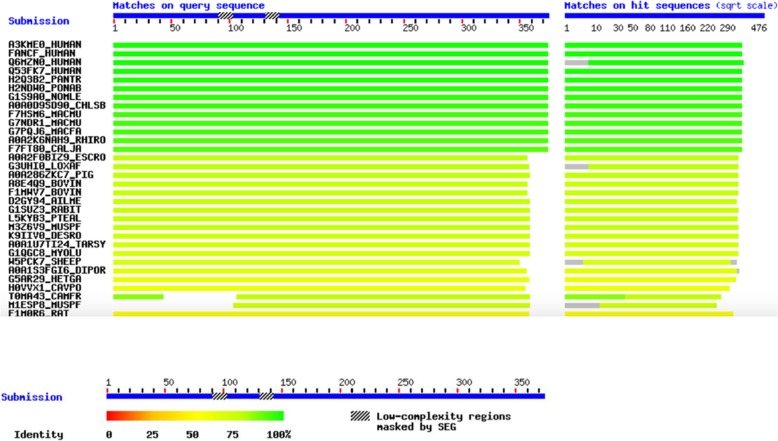
Multiple protein sequence alignment of FANCF across different kingdoms

The comparative amino acid alignment of *FANCF* protein across most kingdoms was also carried out. As shown in Fig. 3, most of the residues were highly conserved during evolution, and any frame shift mutations could be deleterious.

**Fig. 4:**
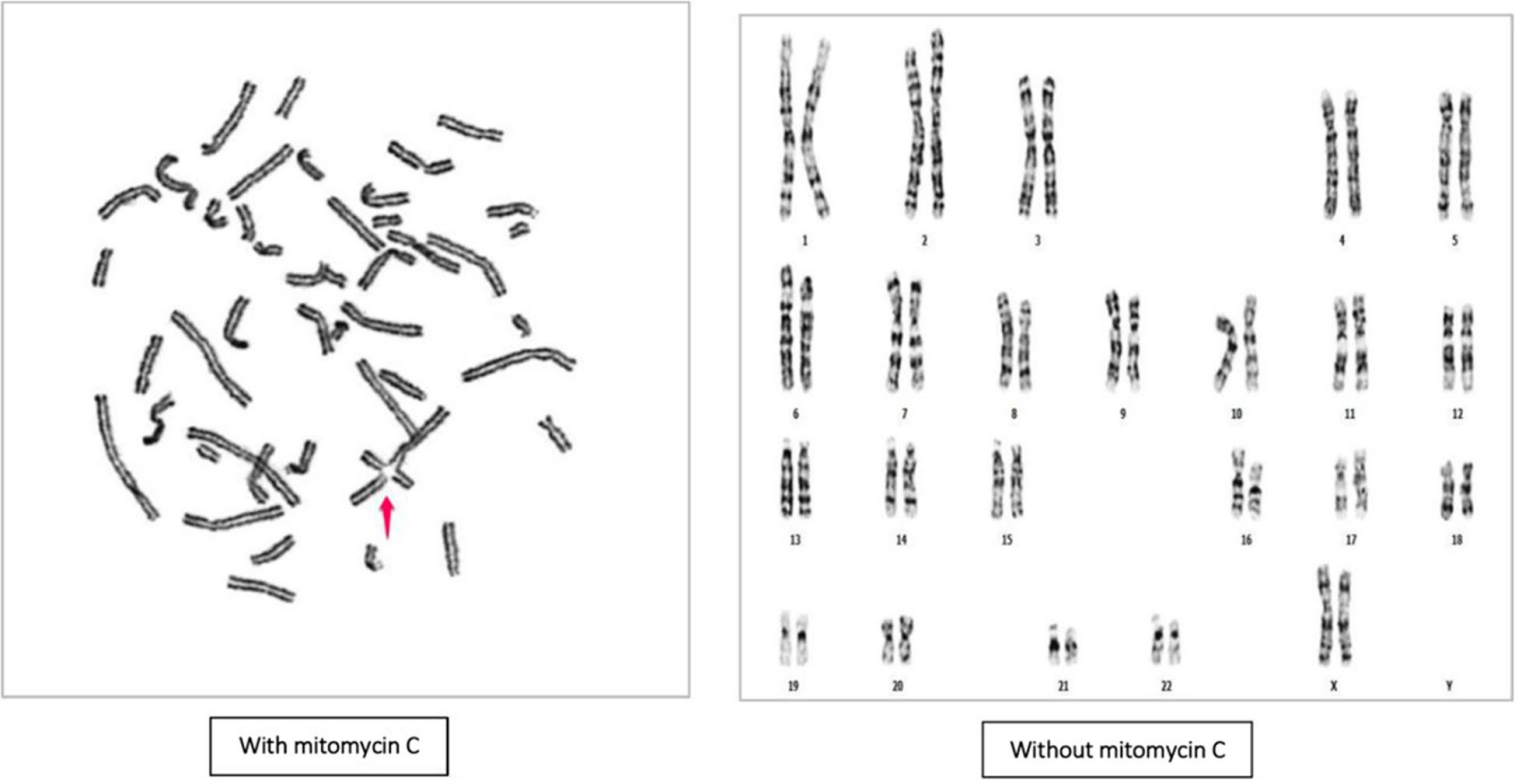
Comparison of GTG binding between blood cultures, exposure to mitomycin C from the proband and normal control without exposure to this agent

In the cytogenetic study, 100 metaphase spreads were studied from cultures prepared by adding MMC and compared to age-related control. The chromosomal breakage scoring was performed on 5 different slides (one untreated with MMC, one with 150 nM MMC concentration, one with 300 nM MMC concentration and one normal control sample treated with these 2 concentrations of MMC). 25 metaphases were evaluated on each slide for chromosomal aberration (gaps or breaks or radial formations). The results showed about 7–8 breaks/cell on average. In comparison to normal control sample which showed 0.3–0.5 breakages/cell. There was no radial formation in the normal control sample. The study showed 46, XX with multiple breaks and radial formation (quadri and triradial), compatible with Fanconi Anemia (Fig. 4, Additional file 1).

## Discussion and conclusion

To date, 22 *FANC* genes have been identified to be mutated in FA patients. The most common genes involved in FA are *BRCA2* (FA-D1, ~ 3%), *BRIP1* (FA-J, ~ 2%), *FANCA* (FA-A, 60–70%), *FANCB* (FA-B, ~ 2%), *FANCC* (FA-C, ~ 14%), *FANCD2* (FA-D2, ~ 3%), *FANCE* (FA-E, ~ 3%), *FANCF* (FA-F, ~ 2%), *FANCG (*FA-G, ~ 10%) and *FANCI* (FA-I,~ 1%). However, less common genes are *ERCC4* (FA-Q), FANCL (FA-L), *FANCM* (FA-M), *MAD2L2* (FA-V), *PALB2* (FA-N), *RAD51* (FA-R), *RAD51C* (FA-O), *RFWD3* (FA-W), *SLX4* (FA-P), UBE2T (FA-T), *XRCC2* (FA-U) [2, 5, 9–12],*MAD2L2 (REV7,FANCV)* [13]*, andRFWD3* [14]*.*

**Fig. 5:**
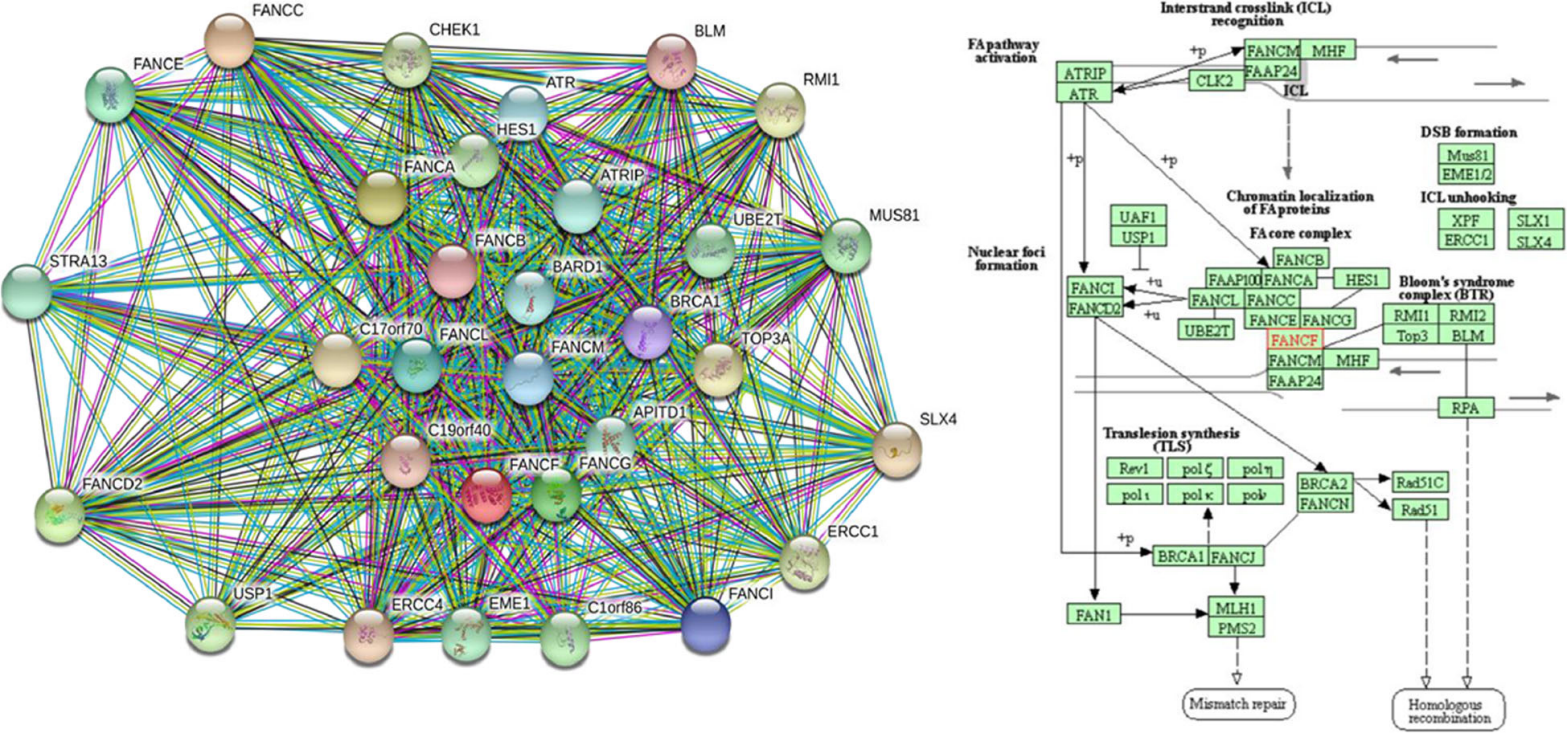
FA Protein network and its association with other proteins extracted from STRING (left) and FA pathway and different involved genes provided from the KEGG database (right)

Although FA Proteins do not have similar sequences, they are correlated with their association and interactions in a common multi subunit protein complex. As shown in Fig. 5, which were extracted from STRING (STRING: functional protein association networks, https://string-db.org) tool and KEGG database (KEGG: Kyoto Encyclopedia of Genes and Genomes, http://www.genome.jp/kegg/), different genes are involved in and interact with FA pathways. It can be expected to identify new genes involved in this disorder as the list of its corresponding genes is growing. Since the FA pathway and its components play a vital role in repairing DNA damage, any impairments of these proteins result in life-threatening abnormalities and hypersensitivity to DNA cross-linking agents, leading to a high frequency of chromosomal instability [1].

In our study, we identified, for the first time, a novel, private, homozygous frame-shift deletion mutation in *FANCF* in an Iranian patient with FA. This gene, located on 11p14.3, is one of the rare genes without intron in the human genome and encodes a DNA repair protein (374 amino acids, 42,254 Da) [15]. The Pfam database indicates that amino acids 1–354 are considered as FANCF domain, while ProDom reported amino acids 1–355 as this domain (https://www.ensembl.org). Therefore, most of the amino acids are essentials for proper function of the protein.

The FANCF gene is the only rarely mutated in FA. At present, 14 different pathogenetic mutations of FANCF account for approximately 2 to 3% of the affected individuals. Disease-associated mutations have been reported throughout the single coding exon of the FANCF gene. The most commonly seen FANCF mutations are short deletions, resulting in frame-shifts and premature termination of the protein. In the study by Nicchia et al. FANCF loss-of-function mutation was associated with a severe phenotype characterized by multiple malformations. FANCF and FANCD2 have been found to be involved in drug-resistant multiple myeloma, ovarian cancer, non-small-cell lung cancer, and head and neck cancer [16–18].

The following evidence can confirm that the identified mutation causes FA in our patient: 1- NGS only identified this mutation to be impaired in the proband and Sanger sequencing confirmed it as homozygous. 2- The novel identified mutation is a frame-shift deletion after the position of 178 in a 374 amino acid protein, coding a fully non-functional truncated protein since most of the amino acids of the FANCF are included in the FANCF domain of this protein. 3- Multiple sequence alignment revealed the conservation of more amino acids of the FANCF during evolution.

FANCF in complex with three FANC proteins, including FANCA, FANCG, and FANCL, interacts with HES1 that is proposed to play a key role in the stability and nuclear localization of the FA core complex proteins. A study conducted by De Winter et al. (2000) [19] identified that FANCF was located primarily in the nucleus, wild-type cells, and a protein complex containing FANCA, FANCC, and FANCG, indicating its role in the maintenance of genomic integrity.

A report of any novel mutation in *FANCF* and other related FA genes can help shed light on the path mechanisms of this disorder and therapeutic strategies, and establishment of genotype-phenotype correlations. A novel, private, homozygous frameshift mutation in the FANCF gene (NM_022725: c. 534delG, p. G178 fs) in our patient correlated with hematological problem (pancytopenia), short stature, microcephaly, and skin hyperpigmentation. Until now, no evidence of malignancy was detected. The authors of this article intend to do more family and functional study on the proband’s relatives. We should also follow the patient for any further somatic mutations or incidence of any type of cancer. If this mutation is reported again in the databases we can make a group and work on genotype-phenotype correlations more practically.

## Additional files

Additional file 1: (Graphical abstract): This image describe and summarize the article in picture version. (TIF 1096 kb)

Additional file 2: (Consent form): Written informed consent form was signed by the patient’s father. (JPG 1497 kb)

## Data Availability

If researchers are willing to access the data, the corresponding author should agree upon providing the interested researcher with the data.

## Abbreviations

DEB: Diepoxybutane
FA: Fanconi anemia
Hb: hemoglobin
MMC: Mitomycin C
MRI: Magnetic resonance imaging
NGS: Next Generation Sequencing
PCR: Polymerase chain reaction
TORCH: Toxoplasmosis, Other (syphilis, varicella-zoster, parvovirus B19), Rubella, Cytomegalovirus, and Herpes
WBC: White blood cells
