# Supplementary figures and images for: A novel frame-shift deletion in FANCF gene causing autosomal recessive Fanconi anemia: a case report
Source: BMC Med Genet. 2019 Jul 9;20:122. doi: 10.1186/s12881-019-0855-2 (PMC6617641; doi:10.1186/s12881-019-0855-2)

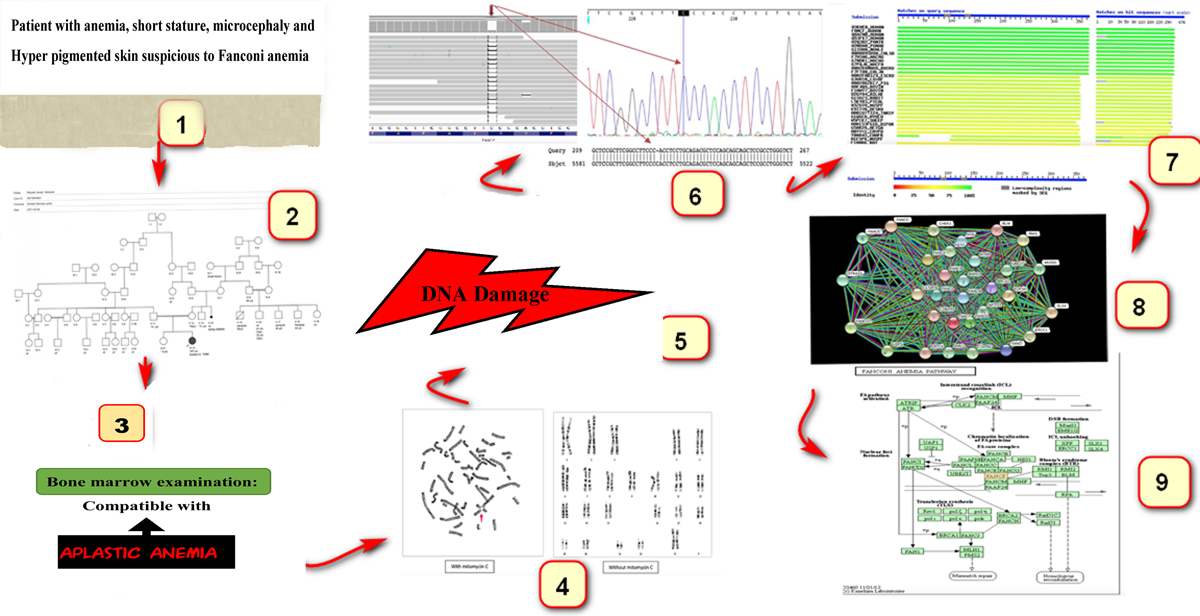

Supplement: Supplementary file 1 — (Graphical abstract): This image describe and summarize the article in picture version. (TIF 1096 kb) [file 12881_2019_855_MOESM1_ESM.tif]

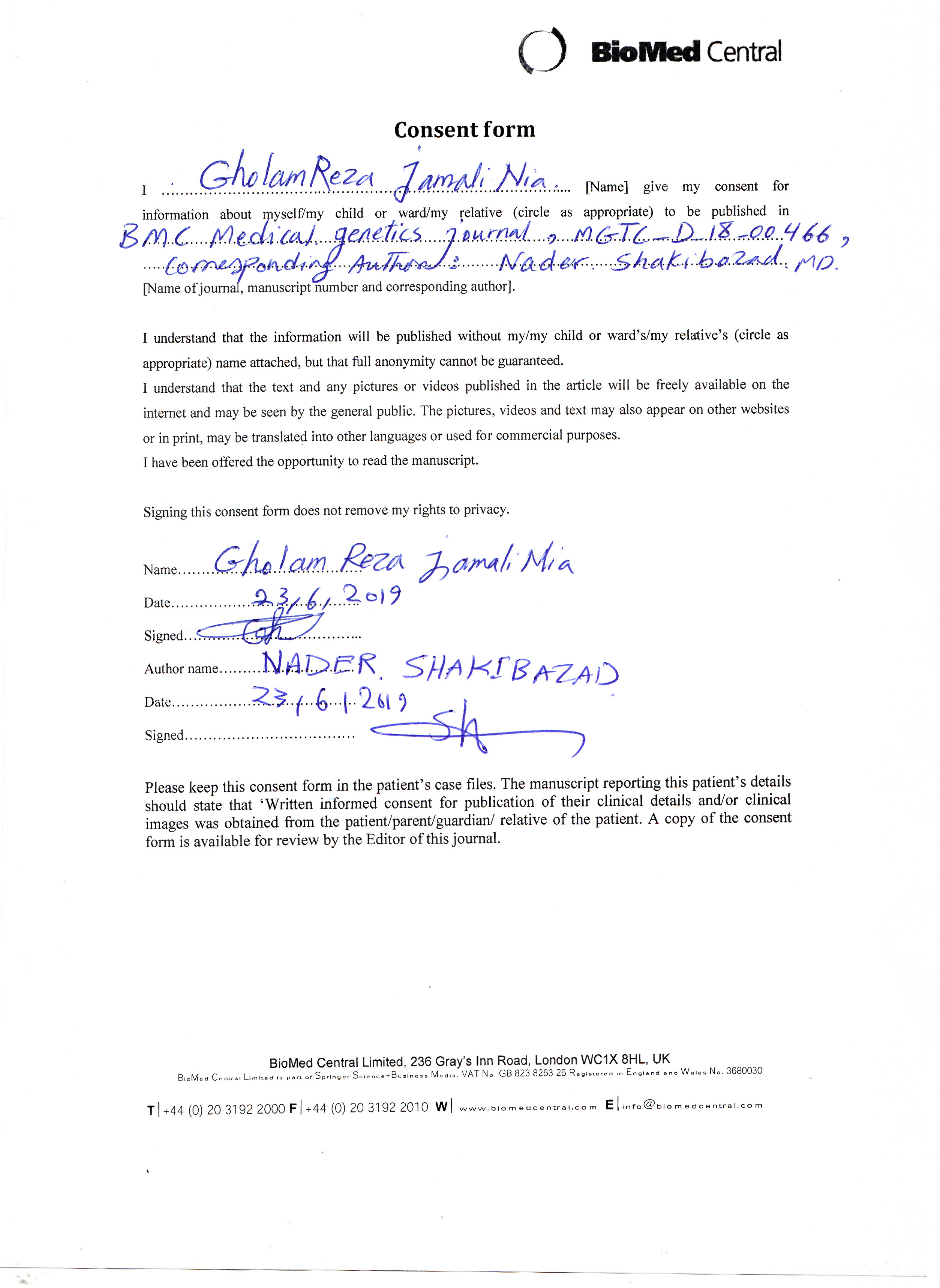

Supplement: Supplementary file 2 — (Consent form): Written informed consent form was signed by the patient’s father. (JPG 1497 kb) [file 12881_2019_855_MOESM2_ESM.jpg]
